# Successful Radiofrequency Ablation of an Atrial Flutter in an Elderly Patient With Uncorrected Ebstein’s Anomaly

**DOI:** 10.7759/cureus.12242

**Published:** 2020-12-23

**Authors:** Ahmad Rimawi, Asem Rimawi

**Affiliations:** 1 School of Medicine, Jordan University Hospital, Amman, JOR; 2 Department of Cardiology, St. Vincet Hospital, Ascension Medical Group, Evansville, USA

**Keywords:** ebstein anomaly, ablation

## Abstract

Ebstein’s anomaly is a rare congenital heart disorder that causes displacement of the tricuspid valve towards the apex of the heart. Patients with this anomaly rarely survive beyond the age of 70 without surgical correction. When such patients survive till adulthood, they tend to present with atrial arrhythmias. Although radiofrequency ablation is harder to successfully complete due to disrupted anatomy of the heart, it can be the best choice for terminating arrhythmias in such patients. Here, we present a case of a 76-year-old patient with an uncorrected Ebstein’s anomaly that presented with atrial flutter and was treated successfully with radiofrequency ablation.

## Introduction

Ebstein’s anomaly is a rare congenital heart disorder characterized by an apical displacement of the tricuspid valve. This disorder accounts for less than 1% of all cases of congenital heart disease [1]. Only 5% of patients with Ebstein’s anomaly reach adulthood and rarely survive till the age of 70 without surgical correction. Atrial arrhythmias are common and may occur in up to one-third of this group [2]. Although radiofrequency catheter ablation is the treatment of choice for such arrhythmias, it is associated with a lower success rate than the normal population and is harder to successfully complete [3]. Here we present a case of an elderly patient with an uncorrected Ebstein’s anomaly that developed an atrial flutter and was successfully treated with radiofrequency catheter ablation.

## Case presentation

**Figure 1:**
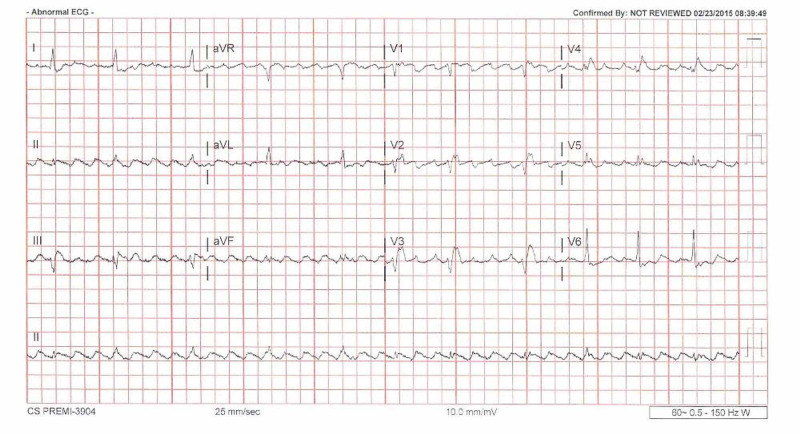
Electrocardiogram showing atrial flutter

A 76-years-old Caucasian man with known Ebstein’s anomaly and coronary artery disease was admitted to the hospital for left-sided chest pain associated with some lightheadedness. Years ago, the patient was evaluated at a tertiary center for Ebstein’s anomaly, and the decision was not to operate at that time because he was unfit for surgery. Upon admission, an electrocardiogram was done and showed atrial flutter (Figure 1).

The patient had negative cardiac enzymes and was hemodynamically stable. Echocardiogram showed reduced left ventricular systolic function, with an estimated ejection fraction (EF) of 20-30%. The tricuspid valve was ventricularly displaced consistent with Ebstein’s anomaly. The next day the patient underwent a transesophageal echocardiogram that showed no left atrial appendage clot. 

After informed consent, the patient underwent an electrophysiological study. Under sedation with midazolam, access was established via the right femoral vein. Upon advancement of the catheter into the heart, it showed that the flutter was right-sided and counterclockwise. We were unable to place the coronary sinus (CS) catheter in place due to an enlarged right atrium (RA) and possible rotation. We placed the CS catheter on the posterior septal region. Entrainment was used to confirm that the flutter was typical and Cavo-tricuspid isthmus (CTI) dependent. Ablation of the CTI was done with conventional mapping only. An 8mm Blazer™ Catheter (Boston Scientific, Marlborough, USA) was used as well as RAMP™ sheath (Abbott, Chicago, USA). Post ablation, the patient had a bidirectional block with lateral RA to the medial (posterior septal) interval was 200 msec and atrial flutter terminated. This was true in the reverse fashion as well. The procedure was completed without any complications, and the patient was discharged on the second day postoperatively. The patient was followed up serially for two years and showed no recurrence of arrhythmias until his death at the end of the second year.

## Discussion

Ebstein’s anomaly is a very rare congenital heart disorder that was first described by Wilhelm Ebstein in 1886 [4]. This anomaly is characterized by the displacement of the tricuspid valve towards the apex of the right ventricle, accompanied by various degrees of tricuspid valve regurgitation. This causes the portion of the right ventricle above the tricuspid valve, along with the right atrium, to dilate [5]. Dilation of the right atrium predispose patients with Ebstein’s anomaly to atrial arrhythmias such as atrial flutter and atrial fibrillation [6]. When this disease presents during adulthood, the most common clinical presentation is supraventricular arrhythmias affecting nearly 30-50% of adults with uncorrected Ebstein's anomaly [7]. 

Radiofrequency catheter ablation is a safe and effective method to treat such arrhythmias. However, in patients with Ebstein’s anomaly, catheter ablation is much harder to successfully complete. This is because the normal anatomy of the right atrium is markedly disrupted due to dilation. In addition to dilation, tricuspid regurgitation will prevent stabilization of the catheter during the procedure, causing failure of the ablation and recurrence of arrhythmias [8]. In this patient, the dilated atrium made it difficult for us to place the catheter in the coronary sinus, and instead, it was placed in the posterior septal region. 

Ebstein’s anomaly is associated with an increased incidence of heart failure and arrhythmias. Only 5% of patients with Ebstein’s anomaly survive till the age of 50 without surgical correction [1]. It is extremely rare to survive beyond the age of 70 with this anomaly uncorrected [9]. Our patient's presentation at this age without the need for surgical correction in his lifetime was very unusual even though he developed severe heart failure from his anomaly. He was also unfit for surgery at the time of diagnosis, preventing him from undergoing surgical correction. Radiofrequency ablation was the most suitable method for his management as it is a minimally invasive procedure that does not require general anesthesia and is associated with a low risk for complications. To the best of our knowledge, this is the first report of an elderly patient with uncorrected Ebstein’s anomaly that developed an atrial flutter and was treated with radiofrequency ablation at such an old age. 

## Conclusions

Radiofrequency ablation is a suitable method to treat supraventricular tachycardias in patients with Ebstein’s anomaly. Although it is harder to complete and associated with an increased recurrence rate compared to the normal population, it is a minimally invasive procedure that is extremely safe, especially in patients that are unfit for cardiac surgery.
